# Comparison of Quantification of Target-Specific Accumulation of [^18^F]F-siPSMA-14 in the HET-CAM Model and in Mice Using PET/MRI

**DOI:** 10.3390/cancers13164007

**Published:** 2021-08-09

**Authors:** Jessica Löffler, Carmen Hamp, Ellen Scheidhauer, Daniel Di Carlo, Christoph Solbach, Alireza Abaei, Li Hao, Gerhard Glatting, Ambros J. Beer, Volker Rasche, Gordon Winter

**Affiliations:** 1 Center for Translational Imaging, Core Facility Small Animal Imaging, Ulm University, 89081 Ulm, Germany; alireza.abaei@uni-ulm.de (A.A.); lihao290310129@gmail.com (L.H.); volker.rasche@uni-ulm.de (V.R.); 2 Department of Nuclear Medicine, Ulm University Medical Faculty, 89081 Ulm, Germany; carmen.hamp@uniklinik-ulm.de (C.H.); ellen.scheidhauer@uniklinik-ulm.de (E.S.); christoph.solbach@uniklinik-ulm.de (C.S.); ambros.beer@uniklinik-ulm.de (A.J.B.); 3 Pharmaceutical Radiochemistry, Technical University of Munich, 85748 Garching, Germany; daniel.di-carlo@tum.de; 4 Department of Nuclear Medicine, Medical Radiation Physics, Ulm University Medical Faculty, 89081 Ulm, Germany; gerhard.glatting@uni-ulm.de; 5 Department of Internal Medicine, Ulm University Medical Faculty, 89081 Ulm, Germany

**Keywords:** PSMA, HET-CAM, chick embryo, PET, MRI, fluoride ligand, prostate cancer, 3Rs principles, in ovo, in vivo

## Abstract

**Simple Summary:**

Animal studies are essential for the development of new radiopharmaceuticals to determine specific accumulation and biodistribution. Alternative models, such as the HET-CAM model, offer the possibility of reducing animal experiments in accordance with the 3Rs principles. Accurate quantification of tumor accumulation of a PSMA-specific ligand in the HET-CAM model and comparison with corresponding animal experiments was performed using the imaging modalities PET and MRI. It was demonstrated that the HET-CAM model leads to comparable results and is suitable as an alternative to animal experiments for the initial assessment of target-specific binding of novel radiopharmaceuticals. However, as evaluation of biodistribution in ovo is still limited, further animal experiments with promising compounds are mandatory.

**Abstract:**

Assessment of biodistribution and specific tumor accumulation is essential for the development of new radiopharmaceuticals and requires animal experiments. The HET-CAM (hens-egg test—chorioallantoic membrane) model can be used in combination with the non-invasive imaging modalities PET and MRI for pre-selection during radiopharmaceutical development to reduce the number of animal experiments required. Critical to the acceptance of this model is the demonstration of the quantifiability and reproducibility of these data compared to the standard animal model. Tumor accumulation and biodistribution of the PSMA-specific radiotracer [^18^F]F-siPSMA-14 was analyzed in the chick embryo and in an immunodeficient mouse model. Evaluation was based on MRI and PET data in both models. γ-counter measurements and histopathological analyses complemented these data. PSMA-specific accumulation of [^18^F]F-siPSMA-14 was successfully demonstrated in the HET-CAM model, similar to the results obtained by mouse model studies. The combination of MR and PET imaging allowed precise quantification of peptide accumulation, initial assessment of biodistribution, and accurate determination of tumor volume. Thus, the use of the HET-CAM model is suitable for the pre-selection of new radiopharmaceuticals and potentially reduces animal testing in line with the 3Rs principles of animal welfare.

## 1. Introduction

Novel radiopharmaceuticals are constantly being developed for diagnosis and treatment of cancer. In the corresponding development processes, information on the target specific accumulation in the tumors as well as the non-specific distribution in the healthy organs is essential to optimize these substances accordingly. Although several of these characterization steps can be performed in vitro, the systemic accumulation and distribution in the body thus far requires an in vivo evaluation, which is primarily performed in small animal models. To reduce or replace the number of necessary laboratory animals in the sense of the 3Rs principles (refinement, reduction, replacement), alternative methods are highly demanded.

Spheroid or 3D organoid models are emerging alternatives that can already partially replicate tumor heterogeneity but are still significantly inferior to real in vivo experiments in terms of complexity and lack of vascularization [1,2].

The Hen’s egg test-chorioallantoic membrane (HET-CAM) model is widely used in translational research and has the potential to bridge between conventional in vitro and in vivo methods, and such, to at least reduce the number of laboratory animals required [3,4,5,6,7,8,9,10,11,12,13,14].

The development of the chick embryo proceeds from the beginning of incubation to hatching within 21 days. The chorioallantoic membrane, as a fusion of the chorion and the allantois, starts to form around the fifth day of embryonic development (EDD) [15,16]. Due to a high vascularization of this membrane as well as a natural immunodeficiency of the chick embryo model [8,17,18,19], tumors can be established on the membrane with modest effort.

Among the major advantages of the chicken egg model are its simple handling, cost efficiency, and significantly reduced maintenance effort compared to small animal models. Further, in accordance with European law (Directive 2010/63/EU of the European Parliament and of the Council of 22 September 2010 on the protection of animals used for scientific purposes), no legal or ethical approvals are required for experiments with the HET-CAM model if sacrificed before hatching. Although in Germany the HET-CAM model is not classified as an animal experiment, for each respective country, the specific legal situation regarding avian embryos must be consulted. Although regulations may slightly differ between countries, the model appears highly attractive for efficient initial screening of new compounds prior to evaluation in small animals.

Although tumors have been already successfully established on the CAM [4,20,21,22,23,24,25,26,27,28,29], and PET-imaging studies are available [10,24,25,30,31,32,33,34,35], systematic analyses of their use to evaluate the biodistribution of radiopharmaceuticals and direct comparisons with the in vivo gold standard are still rare [10].

**Figure 1:**
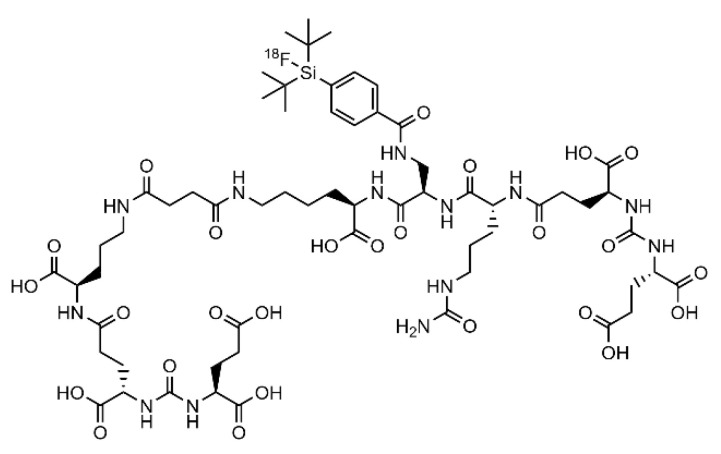
Chemical structure of the PSMA-specific radioligand [^18^F]F-siPSMA-14.

In a previous study on the accumulation of [^68^Ga]Ga-PSMA-11 [24] we demonstrated the potential of the HET-CAM model with respect to analyses of tumor-specific binding of the peptide. In this study, we evaluate the HET-CAM model in direct comparison to an established mouse model regarding biodistribution and specific target binding using the clinically established ^18^F-labelled prostate-specific membrane antigen (PSMA) [36] ligand [^18^F]F-siPSMA-14 (Figure 1) as a model system [37].

## 2. Materials and Methods

### 2.1. Synthesis and Radiolabeling

For radiolabeling of the peptide [^19^F]F-siPSMA-14 (Technical University Munich, Garching, Germany), via isotopic exchange (^18^F/^19^F), fluorine-18 was generated using a PETtrace 860 cyclotron (GE Healthcare, Uppsala, Sweden). Details on this new radiopharmaceutical will be published elsewhere. Synthesis was performed according to the manufacturer’s synthesis instructions (Scintomics, Fürstenfeldbruck, Germany) for the synthesis kit on a GRP cassette module, resulting in [^18^F]F-siPSMA-14 with an average specific activity of A_s_ = (138 ± 50) MBq/µg.

### 2.2. Cell Culture Preparation

The androgen-independent and PSMA-positive (PSMA^+^) prostate carcinoma (PCa) cell line LNCaP C4-2 (ViroMed Laboratories, Minnetonka, MN, USA) [38] and the PSMA-negative (PSMA^−)^ PCa control PC-3 (ACC465, DSMZ, Braunschweig, Germany) [39] were used to establish xenografts in the HET-CAM and mouse models. While LNCaP C4-2 forms highly vascularized tumors with low hypoxia and little rim-core effects [40], PC-3 tumors grow more invasive and are highly proliferating [41], less vascularized, and have a hypoxic and necrotic core [42]. The cell lines were cultivated as described elsewhere [43]. Cells were counted using a Neubauer improved hemocytometer (C-Chip, DHC-N01, NanoEnTek, Seoul, Korea).

### 2.3. HET-CAM Experiments

HET-CAM experiments were performed as described before [24]. In short, the chick embryos were incubated at 37.8 °C and 65% relative humidity starting at embryo development day (EDD) 0. The eggshell was opened on EDD2. Two silicone rings were placed on the CAM on EDD5 and 1 × 10^6^ of PC-3 (PSMA^−^) and 2 × 10^6^ of LNCaP C4-2 (PSMA^+^) tumor cells mixed with Matrigel (30%, v/v) in an overall volume of 45 µL were applied to either ring on EDD6. Tumor growth and embryo health were monitored daily by visual inspection. The MR and PET imaging experiments were performed between EDD13 and EDD15. The chick embryos were cooled for 120 min at 4 °C prior to the MR measurement to prevent motion artefacts, according to the protocols of Bain et al. and Zuo et al. [9,44]. Prior to the PET scan, 150 µL of [^18^F]F-siPSMA-14 ((11.3 ± 0.3) µg/mL) was injected into a chorioallantoic membrane blood vessel through a 30G needle (B. Braun, Melsungen, Germany). An average activity of (2.4 ± 0.9) MBq (median dose 2.4 MBq) was injected. Bleedings after the injection were stopped with cotton buds. The complete chick embryo and needle, syringe, and cotton buds, were measured in an activity meter (CRC-12, Capintec, NJ, USA) to determine the successfully applied radioactivity (100% injected activity [%IA]) for further quantification. In total, 34 chick embryos with tumors were selected for measurements from which 8 (23.5%) had to be excluded due to failed peptide injection.

### 2.4. Animal Studies

The biodistribution of the radioligand was analyzed in male immunodeficient CB17/lcr-Prkdc scid/Crl mice (SCID; *n* = 9; Charles River Laboratories, Sulzfeld, Germany). Similar to the HET-CAM approach, tumor xenografts of the human prostate carcinoma cell lines LNCaP C4-2 and PC-3 were established by administration of 1 × 10^6^ cells subcutaneously into the subscapular regions (left = LNCaP C4-2; right = PC-3) of the SCID mice. The LNCaP C4-2 tumors developed over a period of three weeks after injection. Since the PC-3 tumors grow more rapidly, injection of the PC-3 tumor cells was performed one week after the injection of the LNCaP C4-2 cells.

The mice were anesthetized using 1.5% isoflurane in pressured air/oxygen (80/20%) and a catheter was placed to the tail vein for intravenous injection using 0.9% saline solution containing 5% heparin. The animals were first measured at the MR and then transferred to the PET. After start of the PET measurement, 150 µL of [^18^F]F-siPSMA-14 (11.4 ± 0.3) µg/mL was injected via the catheter with average activity of (3.3 ± 1.6) MBq (median activity 2.8 MBq). During measurements and during transport, the animals were kept continuously anaesthetized. Nine mice were prepared for the studies from which two had to be excluded due to extravasation.

The studies were approved (ethical approval code 1375) by the national authority (Regierungspräsidium Tübingen, Baden-Württemberg) in compliance with German laboratory animal experimentation act and study procedures were in accordance with the European Communities Council Directive of 22 September 2010 (2010/63/EU). All applicable institutional and national guidelines for the care and use of animals were followed.

### 2.5. MRI and PET Measurements

For MRI, precooled chicken eggs were placed in a customized holder. The holder contained fiducials filled with CuSO_4_ and [^18^F]F-siPSMA-14 to facilitate the PET and MR co-registration. MR measurements were based on implemented protocols according to Zuo et al. [9,45]. Data were obtained with a 72 mm quadrature volume T/R resonator on an 11.7 Tesla small animal MRI system (Bruker BioSpec 117/16, Bruker Biospin, Ettlingen, Germany).

For evaluating the ligand biodistribution, a T1-weighted 3D-Flash sequence covering the whole chicken egg was acquired. For accurate assessment of the tumor volume, location, and structure a high resolution T2-weighted multislice rapid acquisition with relaxation enhancement (RARE) sequence was applied. Scan parameters were as: TR/TE = 4320/45 ms, matrix size = 650 × 650, in-plane resolution = 77 × 91 μm^2^, slice thickness = 500 µm, no interslice gap, RARE factor = 8, and NSA = 4. Cover of the whole tumor region required 30 slices, resulting in a 20 min acquisition time.

Anatomic images of the mouse were obtained with a multislice FLASH sequence with acquisition parameters as: TR/TE = 150/1.5 ms, flip angle FA = 15°, matrix size = 750 × 300, in-plane resolution = 100 × 133 µm^2^, slice thickness = 500 µm, and NSA = 12.

To assess the biodistribution of [^18^F]F-siPSMA-14 in chick embryos and in mice, a dynamic 60 min scan was performed using a small animal PET scanner (Focus120, Siemens Medical Solutions, Inc., Erlangen, Germany). The Focus120 has a high spatial resolution (<1.3 mm) and high sensitivity (approximately 7%) with a bore size of 12 cm diameter and 7.6 cm axial length [46]. Obtained list mode files were processed to create histogrammed data (sinograms) for an image of 12 dynamic frames of 5 min each. Reconstructions were performed applying OSEM3D/MAP using 4 OSEM2D, 2 OSEM3D, and 18 MAP iterations with a matrix of 256 × 256 and a zoom factor of 1.5.

MRI and PET of chick embryo and mice were either fused by fiducial registration using 3Dslicer (ver. 4.11.20210226) [47] or by automatic rigid overlay using the software tool PMOD (PMOD Technologies, Zürich, Switzerland). Additional data conversion was achieved using the Vinci software (ver. 5.06) [48].

In the HET-CAM model volumes-of-interest (VOIs) were identified manually in the MR images for the LNCaP C4-2 and PC-3 tumors as well as for the heart, liver, and brain. In the developing chick embryo, the kidney area was rather difficult to distinguish from the surrounding tissue in the MRI, thus a standardized spherical VOI (3 × 1.5 × 1.5 mm) was placed in the kidney region based on the PET images. An additional VOI (4 × 4 × 4 mm) was positioned in the area of the allantoic fluid to measure possible excretion and the results were added to the supplementary.

In mice, VOIs were manually identified in the MRI in the tumors (LNCaP C4-2 and PC-3), heart, lung, liver, spleen, and kidneys. For the brain region a standardized spherical VOI (3 × 2 × 3 mm) was placed based on the PET images.

Data were decay corrected relative to the time of injection. Time activity curves (TAC) were generated over all PET data (HET-CAM *n* = 26; mice *n* = 7) using Graphpad Prism ver. 9.2.0 (GraphPad Software, San Diego, CA, USA). To compare the TAC, simple linear regressions of HET-CAM and mouse data were performed, starting 10 min after injection, and the slopes were calculated. For each individual pair of tumors, the ratios of the activity concentrations of the tumors (PSMA^+^/PSMA^−^) were calculated.

### 2.6. Ex Vivo Validation

Excised HET-CAM tumor xenografts were washed to reduce overestimation of accumulated activity due to blood spillage. Following a 1 min wash using PBS, the extracted tumors of the HET-CAM models were analyzed by γ-counter COBRA II (Perkin Elmer, Waltham, MA, USA) to accurately quantify the accumulated radioactivity. Mouse tumors were also rinsed to remove blood from the extraction. Tumor volume (mL) was determined for the HET-CAM and mouse model based on the MR images while, in addition, tumor wet weight (g) was measured for the mouse model. Quantification of the radioactivity in the tumor was based on decay-corrected γ-counter data in relation to the total activity injected into the chicken egg or mouse (percent injected activity, %IA). These data were normalized to the MRI-derived tumor volume or tumor wet weight. For the determined activity concentrations in [%IA/mL] or [%IA/g], the mean value ± standard deviation and, additionally, the median were reported in the sections below. The ratio of the activity concentration for each pair of PSMA^+^ and PSMA^−^ xenografts was calculated to provide a measure for evaluation of the relative ligand accumulation. Values > 1 indicate a higher accumulation in the PSMA^+^ tumor.

### 2.7. Protein Expression and Histopathological Analyses

PSMA expression was determined in tumor cell lysates before grafting onto the CAM and tumor lysates after γ-counter measurements. Cells were washed with cold PBS and resuspended in RIPA buffer (Sigma-Aldrich, Taufkirchen, Germany) containing 1× protease inhibitor cocktail (Roche, Basel, Switzerland). LNCaP C4-2 and PC-3 tumors were homogenized in RIPA buffer with 1× protease inhibitor cocktail and incubated on ice for 20 min. Protein concentration was determined using Coomassie Protein Assay Reagent (Thermo Fisher Scientific, Waltham, MA, USA) according to manufacturer’s instructions at a wavelength of 595 nm. Respectively, 20 µg of protein was separated by SDS-PAGE (12% reducing acrylamide gel) and transferred to a nitrocellulose membrane (GE Healthcare, Solingen, Germany). To detect PSMA and glyceraldehyde-3-phophate dehydrogenase (GAPDH), the following antibodies were used: anti-PSMA (abcam, Cambridge, UK, catalog ab19071, 1:500) and anti-GAPDH (Santa Cruz Biotechnology, Inc., Dallas, TX, USA, catalogue SC-365062, 1:2000). As positive control, 0.11 µg recombinant human PSMA (rhPSMA) was used (R&D Systems, Minneapolis, MN, USA).

Tumors were fixed over night with 4% formaldehyde solution in phosphate buffered saline (PBS) pH 7.4 (Thermo Fisher Scientific, Waltham, MA, USA). Tissue was dehydrated and paraffin embedded before preparation of 4 µm sections using a rotary microtome (Leica JUNG RM2045, Wetzlar, Germany). After deparaffinization, antigen retrieval was performed using antigen unmasking solution (BIOZOL, Eching, Germany) for 10 min at 95 °C. To quench endogenous peroxidase activity, sections were treated with 0.75% hydrogen peroxide for 10 min. Subsequently, slides were blocked for 20 min using blocking-serum (BIOZOL). Anti-PSMA antibody (abcam, Cambridge, UK catalog ab133579, 1:800) was incubated over night at 4 °C. The next day, sections were washed with PBS and incubated with biotinylated second antibody (BIOZOL) for 1 h. After another washing step with PBS, sections were treated with the avidin/biotin-based VECTASTAIN^®^ Elite^®^ ABC Reagent (HRP) (BIOZOL) for 40 min. Afterward, slides were washed again and the HRP substrate, Vector^®^ DAB Peroxidase Substrate (BIOZOL), was added for 6 min. DAB substrate turnover was stopped by washing with PBS. Cell nuclei were counterstained with hematoxylin (Waldeck GmbH & Co. KG, Münster, Germany) for 1 min. Following, slides were mounted using Entellan^®^ (Merck, Darmstadt, Germany).

For HE-staining, slices were deparaffinized and cell nuclei were stained with hematoxylin (Waldeck GmbH & Co. KG, Münster, Germany) for 10 min. Cell bodies were stained using a 2% eosin (Waldeck GmbH & Co. KG, Münster, Germany) solution in water for 2 min. Subsequently, slides were mounted in Entellan^®^ (Merck, Darmstadt, Germany).

Images of the section were captured using an Axioskop 2 plus in combination with the AxioCamMRC-TV2/3’’C (Zeiss, Oberkochen, Germany) with 5× magnification lens for overview images and 10× magnification lens for close-up images.

### 2.8. Statistical Evaluation

Wilcoxon matched-pairs signed rank test, Pearson correlation analysis, and simple linear regression were performed using GraphPad Prism (ver. 9.2.0 for Windows, GraphPad Software, San Diego, CA, USA). A *p* value < 0.05 was assumed statistically significant.

## 3. Results

### 3.1. Tumor Growth and Peptide Accumulation in the Tumors

**Figure 2:**
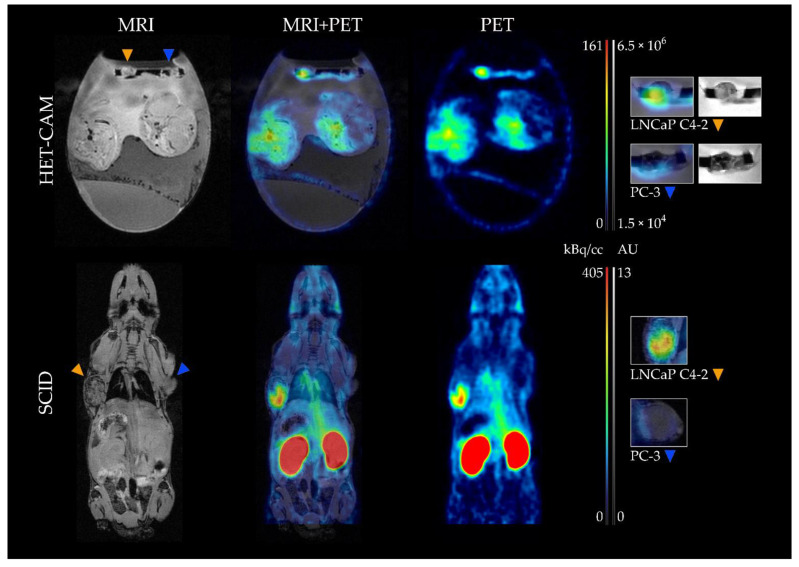
Representative MR and PET images of chick embryo and mice with corresponding fusion image. Anatomy of the chick embryo and mice was obtained by a T1-weighted Flash scan (**left**) while a static reconstruction of a 60 min PET scan was used to demonstrate the biodistribution of the radioligand (**right**). In the resulting fusion image (**middle**), clear accumulation of [^18^F]F-siPSMA-14 could be localized in the PSMA-positive tumor LNCaP C4-2 (orange arrow) while for PC-3 (blue arrow), a weak signal was observed for both in vivo models. Magnified views of the corresponding tumor regions are depicted on the right. For the HET-CAM model, the images were based on the T2-weighted RARE scan and for the mice, on the T1-weighted Flash images. The PET signal in the HET-CAM model is clearly localized in the LNCaP C4-2 tumor, whereas the weak signal slightly below the PC-3 tumor was caused by an adjacent blood vessel.

MRI measurements were used to evaluate tumor growth in the chick embryo model and to accurately determine tumor volumes (Figure 2). The measurements revealed tumor volumes of (0.025 ± 0.008) mL for LNCaP C4-2 and (0.023 ± 0.011) mL for PC-3 after 8 days of tumor growth.

Tumor volumes in the mouse model resulted in (0.22 ± 0.13) mL for LNCaP C4-2 and (0.22 ± 0.10) mL for PC-3 cell lines. The weight of the extracted tumor of (0.27 ± 0.18) g for LNCaP C4-2 (after 21 days of tumor growth) and (0.24 ± 0.12) g for PC-3 (after 14 days of tumor growths) was in good agreement with the MRI-based volumes (LNCaP C4-2 *r* = 0.93; *p* < 0.005/PC-3 *r* = 0.88; *p* < 0.05).

MRI and PET images were successfully obtained as described and overlay of the images using PMOD software allowed the direct correlation of the measured radioactivity to an anatomical region. For the HET-CAM-model as well as for the mouse model, marked accumulation of the radioligand was observed in the PSMA^+^ LNCaP C4-2 tumors. Only weak signals were detected for the PSMA^-^ PC-3 tumor xenografts for both models (Figure 2).

**Figure 3:**
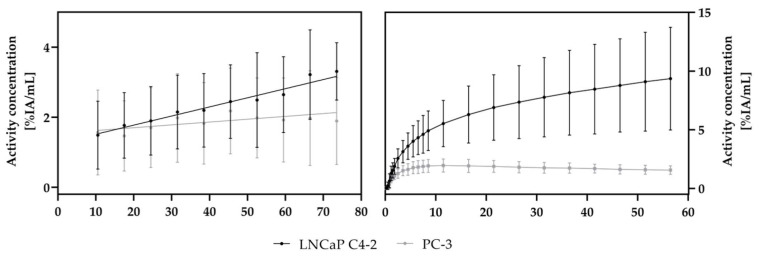
PET-data-based time-activity curves of [^18^F]F-siPSMA-14 accumulation in tumor xenografts of the HET-CAM model (**left**) and the mouse model (**right**). An increasing activity concentration [%IA/mL] over 60 min scan time was observed for the PSMA^+^ LNCaP C4-2 tumors in both models, whereas for PC-3 the signal was roughly constant. The resulting positive slope for LNCaP C4-2 indicates uptake of [^18^F]F-siPSMA-14. VOIs were drawn on anatomical MR scans in the PET/MR fusion images. Depicted mean and standard deviation values were determined using all measured PET data (HET-CAM *n* = 26; mice *n* = 7). Linear regression lines are shown for the HET-CAM data (**left**), whereas connecting lines are shown for the mouse data (**right**).

The TACs based on dynamic PET scans are depicted in Figure 3. Data from all PET scans were used for this analysis. For the HET-CAM model as well as for the mouse model, an accumulation of [^18^F]F-siPSMA-14 was observed to increase with time for the PSMA^+^ LNCaP C4-2 tumor while for the PSMA^-^ PC-3 tumor, a constantly lower level was observed.

In the HET-CAM model, a steady increase (slope: 0.026 ± 0.004) of the activity concentration was observed in the LNCaP C4-2 tumor, where for the PC-3 tumor, a nearly constant signal (slope: 0.008 ± 0.004) resulted.

In mice, a small perfusion peak was observed for PC-3 after catheter injection, followed by a decreasing activity concentration. For the LNCaP C4-2 tumor, a slope of 0.081 ± 0.027 was observed starting 10 min after injection, where in the PC-3 tumor, a slope of −0.009 ± 0.004 resulted based on linear regression.

A clear difference of the TACs between the PSMA^+^ and the PSMA^−^ tumors indicated a PSMA-specific accumulation of [^18^F]F-siPSMA-14 as shown in Figure 3.

**Figure 4:**
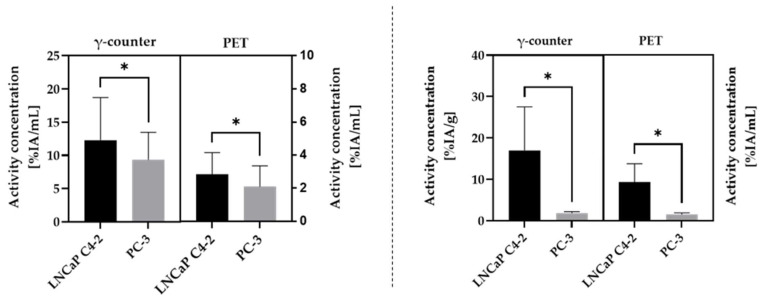
Comparison of chick embryo (**left** **diagram**) and mouse model (**right** **diagram**) using two different evaluation methods (γ-counter and PET-based data). A significantly higher activity concentration (*, *p* < 0.05) in the PSMA^+^ tumor xenograft LNCaP C4-2 is demonstrated regarding PSMA^−^ PC-3 for both evaluation methods and in both in vivo models. Significance was tested by Wilcoxon matched-pairs signed rank test.

For the HET-CAM model, a significantly higher accumulation (*p* < 0.05) in the PSMA^+^ tumor LNCaP C4-2 (mean (12.3 ± 6.4)%IA/mL; median: 11.0%IA/mL) compared to the PSMA^−^ tumor PC-3 (mean: (9.3 ± 4.1)%IA/mL; median: 8.4%IA/mL) was observed for γ-counter measurements (Figure 4), resulting in a ratio of PSMA^+^/PSMA^−^ of 1.5 ± 0.8. (7/26 chick embryos show PSMA^+^/PSMA^−^ ratios < 1). Similar results were obtained comparing the PET values at 60 min post scan start: PSMA^+^ LNCaP C4-2 (mean: (2.8 ± 1.3)%IA/mL; median: 2.61%IA/mL), PSMA^−^ PC-3 (mean: (2.1 ± 1.2)%IA/mL; median: 1.8%IA/mL), resulting in a ratio of PSMA^+^/PSMA^−^ of 2.0 ± 1.5 (Figure 4). 6 out of 26 chick embryos showed PSMA^+^/PSMA^−^ ratios < 1; thus, indicating a specific accumulation of [^18^F]F-siPSMA-14 in the PSMA^+^ tumor LNCaP C4-2.

In the mouse model, a significantly higher accumulation (*p* < 0.05) of [^18^F]F-siPSMA-14 was determined by γ-counter measurements for LNCaP C4-2 (mean: (17.0 ± 10.5)%IA/g; median: 13.6%IA/g) compared to the PSMA^−^ PC-3 (mean: (1.8 ± 0.4)%IA/g; median: 1.8%IA/g) (Figure 4), resulting in a ratio of PSMA^+^/PSMA^-^ of 9.5 ± 5.1. Evaluation from PET data using the last value of the dynamic 60-min PET scans provided similar results: PSMA^+^ LNCaP C4-2 (mean: (9.4 ± 4.4) %IA/mL; median: 8.2%IA/mL); PSMA^−^ PC-3 (mean: (1.6 ± 0.4)%IA/mL; median: 1.6%IA/mL); ratio PSMA^+^/PSMA^−^: 6.4 ± 3.2 (Figure 4).

For both evaluation methods, an increased [^18^F]F-siPSMA-14 uptake in the LNCaP C4-2 tumor was observed in the HET-CAM (1.5-fold higher (γ-counter), 2-fold higher (PET) activity concentration) and the mouse model (9.5-fold higher (γ-counter) and 6.4-fold higher (PET) activity concentration). All data (mean ± SD and ratio) are summarized in Table 1. Calculated activity concentrations, ratios, and tumor volumes for each separate chicken egg and mouse are listed in Tables S1 and S2.

### 3.2. Biodistribution

Combination of high-resolution MR imaging and dynamic PET imaging using [^18^F]F-siPSMA-14 enabled the assessment of biodistribution in organs of interest in chick embryos as well as in mice. Based on fusion images of MRI and PET, time-activity curves were generated and the biodistribution of the radioligand in the chick embryo model and in the mouse model was evaluated.

**Figure 5:**
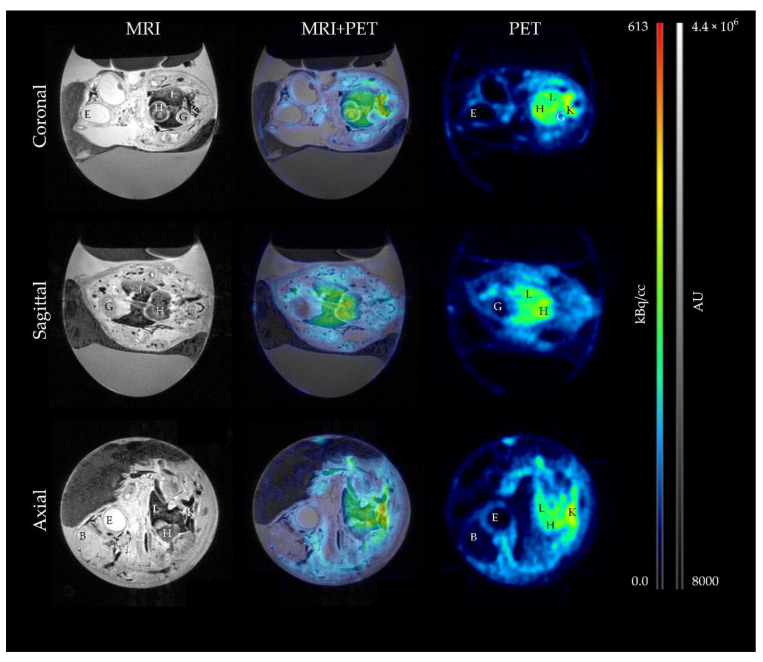
Biodistribution of [^18^F]F-siPSMA-14 in the HET-CAM model visualized by MR and PET imaging. Using anatomical T1-weighted MRI (**left column**), organs can be differentiated in the chick embryo. In the PET images (**right column**), the distribution of [^18^F]F-siPSMA-14 is visualized. By superimposing MRI and PET, the accumulation can be assigned to the respective organs. In the different sectional planes (coronal, sagittal, and axial, where axial was defined to be the main axis in the chicken egg), it is evident that there is no signal in the eye (E), brain (B), or gizzard (G). Accumulation of radioligand is observable in liver (L) and heart (H) while the most intense signal is observable in the kidneys (K). PET signal is presented in kBq/cc and MRI intensity in arbitrary units (AU).

For chick embryo imaging, the main axis of the chicken egg was defined as axial and images from all three axes: coronal, sagittal, and axial, were displayed for better visualization. In the MR image, the following organs could be differentiated as: heart, brain, liver, kidneys, gizzard, and eye. Based on the visual evaluation, most radioactivity was detected in kidneys, heart, and liver. No accumulation of [^18^F]F-siPSMA-14 was detected by visual inspection in brain, gizzard, and eyes (Figure 5).

**Figure 6:**
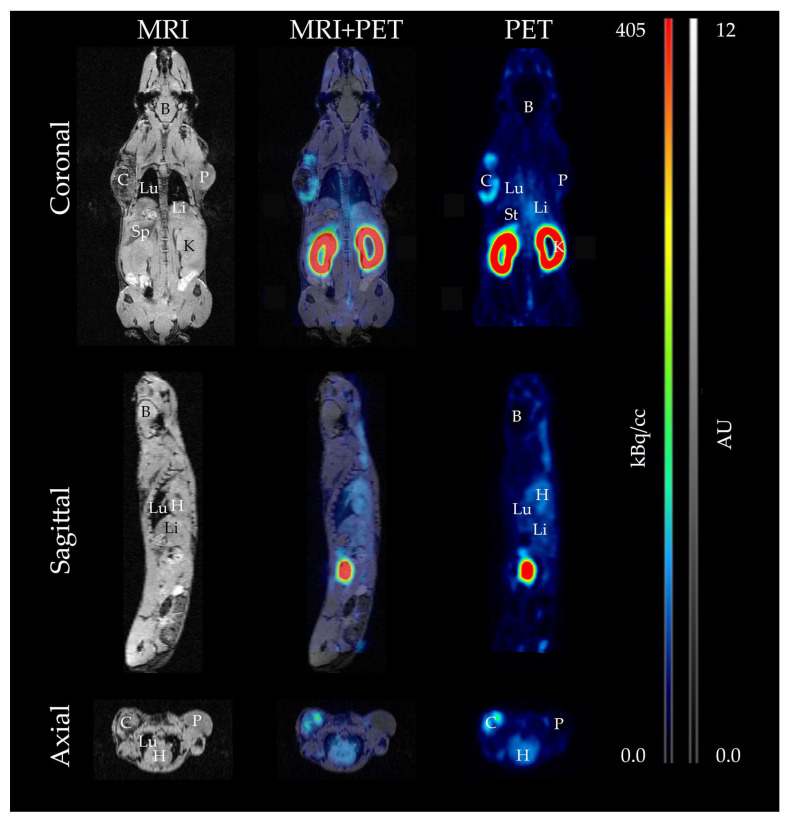
Assessment of [^18^F]F-siPSMA-14 biodistribution in tumor-bearing mice by MRI and PET. Anatomy is visualized by T1-weighted MR images (**left** **panel**), and the accumulation of [^18^F]F-siPSMA-14 is represented by PET images (**right** **panel**). In the corresponding fusion image (**middle column**), the radioactive signal can be assigned to the respective organs. In the 3 section planes (coronal, sagittal, and axial), radioactive signal is assigned in PSMA^+^ LNCaP C4-2 tumor (C), whereas no accumulation higher than the background signal can be seen in PSMA^-^ PC-3 tumor (P). Also, no accumulation of [^18^F]F-siPSMA-14 is detected in brain (B) and stomach (St). Increased activity is observed in heart (H), liver (Li), lung (Lu), and spleen (Sp), whereas the highest activity is detected in the kidneys (K).

In the mouse model, differentiation of the following organs was possible based on MR imaging: heart, brain, liver, kidneys, lung, spleen, and stomach. Highest PET signal was observed in the kidneys, specifically the renal cortex. While a higher signal was also detected for the spleen, no increased accumulation was detected for the other organs. Significantly lower activities were observed for the brain and stomach (Figure 6).

**Figure 7:**
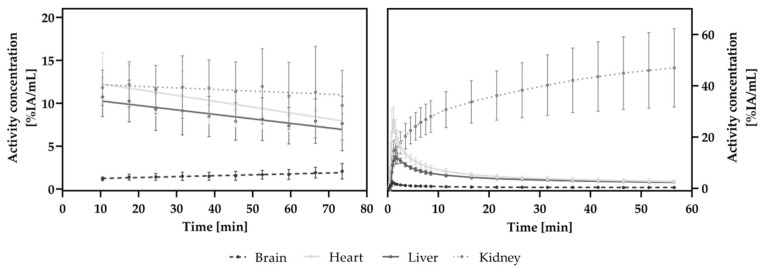
Time-activity curves based on the analysis of PET data of the HET-CAM (**left**) and mouse (**right**) models. The activity concentration of the organs-of-interest (brain, heart, liver, and kidneys) was monitored over a 60 min PET scan. In both models, only low levels of radioactivity are seen in the brain. For heart and liver, a decrease in signal is observed after an initial increase. As measurements in the HET-CAM model were not started until approximately 10 min after injection, there is no increase mapped in the graph. Heart and liver are often correlated with the blood pool. The activity concentration in the kidneys of the chick embryos remained constant, whereas in the mouse model, the activity concentration increased during the measurement.

Time-activity curves for the selected organs: brain, heart, liver, and kidneys, based on the MRI and PET fusion images, are depicted in Figure 7.

In both models, as expected, almost no activity concentration was detected in the brain. The slopes obtained were −0.0006 ± 0.0007 in the mouse model and 0.011 ± 0.002 in the chick embryo. In both the chick embryo and the mouse model, a signal from [^18^F]F-siPSMA-14 was detected in the heart and liver, which decreased considerably over the measurement period. The slopes were similar for these organs in both models with −0.068 ± 0.008 (chick embryo) and −0.078 ± 0.007 (mouse) for heart and −0.052 ± 0.008 (chick embryo) and −0.056 ± 0.005 (mouse) for liver. In the mouse model, due to the catheter injection of the radioligand, a perfusion peak was evident for the heart, brain, and liver in the first minutes after injection.

The highest activity concentration was measured for the kidneys in both in vivo models. Whereas in the HET-CAM model, the activity concentration in the kidneys slightly decreased over time (slope − 0.02 ± 0.01); a considerable increase was detected in the kidneys in the mouse model (slope 0.36 ± 0.09).

For the chick embryo model, the highest activity concentration after 60 min PET measurement was determined in the kidneys (10.8 ± 4.2) %IA/mL, followed by the heart region (8.5 ± 2.0) %IA/mL and the liver (7.4 ± 2.0) %IA/mL. In the brain, the lowest signal (1.8 ± 0.5) %IA/mL was measured, indicating that the ligand cannot cross the blood-brain barrier. Minimal accumulation was also detected in the chick embryo eye (1.8 ± 0.7) %IA/mL; these data were not included in the direct comparison with the mouse model.

Analysis of Pearson correlation between the heart signal and the other organs revealed a strong correlation to liver (*r* = 0.92, *p* < 0.0001), moderate correlation to kidneys (*r* = 0.56, *p* < 0.0001), and a weak correlation to eye (*r* = 0.36, *p* < 0.0001), brain (*r* = 0.33, *p* < 0.0001), PC-3 (*r* = 0.31, *p* < 0.0001), and LNCaP C4-2 (*r* = 0.21, *p* < 0.0005).

**Figure 8:**
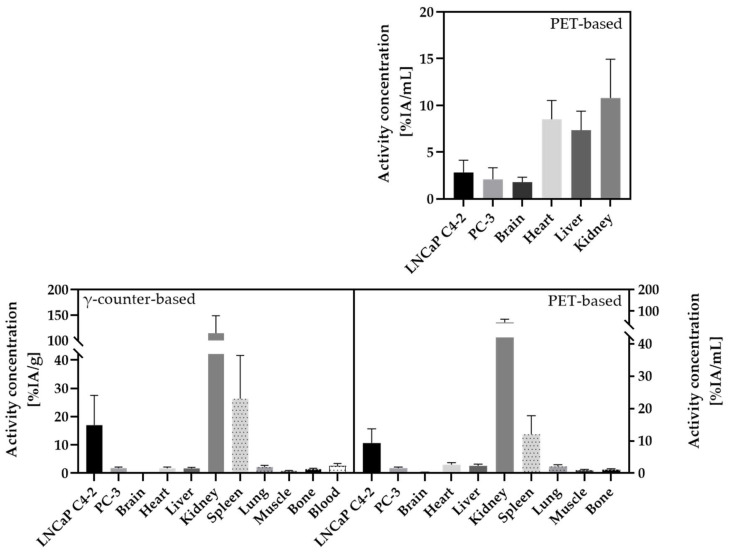
Biodistribution of [^18^F]F-siPSMA-14 in organs of interest, based on PET evaluations in the HET-CAM model (**top** **row**) and PET and γ-counter evaluation in the mouse model (**bottom** **row**), respectively. In both models, as described previously, a significantly higher uptake of [^18^F]F-siPSMA-14 was detected in the PSMA^+^ tumor LNCaP C4-2 compared with the PSMA^−^ tumor PC-3. The highest activity concentration was detected in kidneys in both models, or evaluation approaches. In the mouse, a high activity concentration was additionally detected in the spleen. In both in vivo models, accumulation in the brain was minimal. Low signal was also detected in the heart, lung, muscle, and bone in the mouse with both evaluation methods, whereas based on the PET data in the HET-CAM, activity concentrations were raised in the heart and liver.

Quantification of the distribution of [^18^F]F-siPSMA-14 based on PET data was also performed in the mouse model. At the end of the 60-min PET scan, only modest activity concentrations were detected in brain (0.3 ± 0.1) %IA/mL, muscle (0.9 ± 0.2) %IA/mL, and bone (1.1 ± 0.3) %IA/mL. Higher activity accumulations were detected for heart (2.6 ± 0.6) %IA/mL, liver (2.3 ± 0.5) %IA/mL, and lung (2.1 ± 0.5) %IA/mL. In addition to the high accumulation in the PSMA^+^ tumor (9.4 ± 4.4) %IA/mL as described above, a high value (12.1 ± 5.7) %IA/mL was also detected for the spleen. The peak signal was determined for the kidneys (47.0 ± 15.3) %IA/mL (Figure 8).

The described findings were confirmed by γ-counter-based analysis (Figure 8). Minor accumulations were determined in brain (0.1 ± 0.1) %IA/g, muscle (0.8 ± 0.2) %IA/g, and bone (1.5 ± 0.3) %IA/g. Slightly lower activity concentrations were determined in heart (1.7 ± 0.4) %IA/g and liver (1.7 ± 0.4) %IA/g compared with PET measurement. The value for the lung (2.2 ± 0.6) %IA/g was in close agreement with the PET signal. In the blood, a value of (2.5 ± 0.9) %IA/g was still detectable after 1h. The high activity levels in PSMA^+^ LNCaP C4-2 tumor (17.0 ± 10.5) %IA/g and spleen (26.3 ± 15.3) %IA/g were confirmed by γ-counter measurements. The highest signal, as in the mouse PET and HET-CAM models, respectively, was determined for the kidneys (114.7 ± 34.2) %IA/g. All data (mean ± SD and ratio) were summarized in Table 2. Calculated activity concentrations of the respective organs for each separate chicken egg and mouse are listed in Tables S3 and S4.

Pearson correlation analysis on PET data, comparing the heart signal to the other organs, resulted in strong correlation to lung (*r* = 0.99, *p* < 0.0001), brain (*r* = 0.96, *p* < 0.0001), and liver (*r* = 0.81, *p* < 0.0001), no or weak negative correlation to PC-3 (*r* = −0.26, *p* < 0.001), muscle (*r* = −0.33, *p* < 0.0001), and spleen (*r* = −0.36, *p* < 0.0001), and moderate negative correlation to kidneys (*r* = −0.43, *p* < 0.0001) and LNCaP C4-2 (*r* = −0.50, *p* < 0.0001).

### 3.3. Evaluation of PSMA-Expression

**Figure 9:**
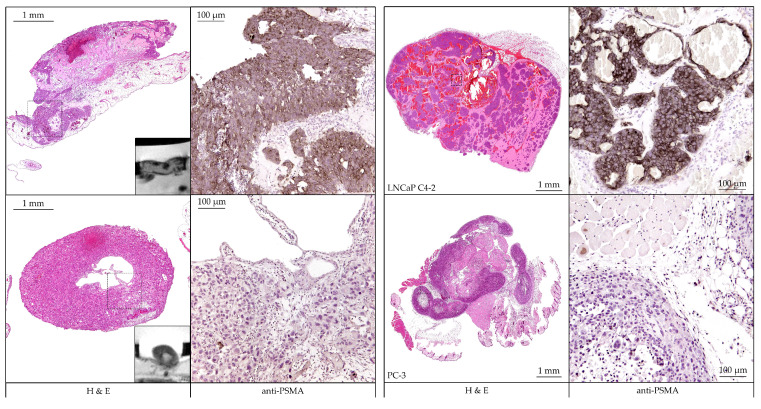
Histopathological analysis of the tumor xenografts grown on the HET-CAM (**left**) and mouse model (**right**). The prostate cancer cell line, based tumors LNCaP C4-2 (**upper** **row**) and PC-3 (**bottom** **row**), were stained using hematoxylin and eosin (H&E) as depicted in overview sections (**left side**). Consecutive sections of the same tumors were stained using a PSMA-specific antibody. Detailed images of the section (regions marked with dotted line boxes) are depicted on the right side. Clearly PSMA-specific staining was observed for LNCaP C4-2 but not for PC-3. MR images of the HET-CAM tumors were imaged in addition to the H&E sections to illustrate the excellent resolution of the RARE sequence.

Immunohistochemistry (Figure 9) in the form of H&E staining and labeling with PSMA-specific antibody were performed to validate tumor growth and target protein expression in the tumor xenograft. Detection staining was performed on consecutive sections in both HET-CAM and mouse models. The tumors are clearly visible in the H&E overview sections and specific staining was clearly detected for the PSMA^+^ tumor LNCaP C4-2 in the magnified sections. Accordingly, no expression was detected in the PSMA^−^ tumor PC-3.

The MR images of the respective HET-CAM tumors additional to the overviews demonstrate the high level of detail of the MR measurements (Figure 9).

PSMA expression was additionally detected by WB (Figure S1). A clear signal was observed in the PSMA^+^ tumor, whereas the low signal in PC-3 was due to non-specific binding of the antibody. Additional detection of the housekeeping protein GAPDH demonstrated that equal amounts of protein were used in all analyses.

## 4. Discussion

In the present comparative study, we successfully demonstrated that the chick embryo model in combination with PET and MR imaging is qualified for the evaluation of specific target binding of radiopharmaceuticals and provides similar results compared to the mouse model using [^18^F]F-siPSMA-14 as a model compound. While we demonstrated that initial information on biodistribution in organs is also possible in the HET-CAM model and in good agreement with the mouse data, further small animal studies for more detailed assessment of biodistribution and dosimetry are still necessary.

Thus, our results corroborate that the PET and MR imaging in the HET-CAM model can be used for initial evaluation of target specific binding of novel radiopharmaceuticals instead of mouse xenograft experiments, which in terms of the 3Rs principles, contributes to the reduction of animal experiments.

### 4.1. Evaluation of Tumor Accumulation of [^18^F]F-siPSMA-14 in the HET-CAM Model Compared to a Mouse Model

We chose the prostate cancer model for our experiments as it is of great clinical relevance and PSMA-specific ligands as well as established cell lines are widely available. The radioligand [^18^F]F-siPSMA-14 was commercially available and has already been used in clinical applications [37].

Based on the activity concentrations and ratios between the PSMA^+^ and PSMA^−^ tumors, kinetics, and correlation analyses, there was evidence of significantly higher accumulation of the ligand in the PSMA^+^ tumor. The results were in good agreement with the data from the mouse experiments. Thus, the HET-CAM model is appropriate for the analysis of specific tumor accumulation based on PET and MR imaging and may contribute to the reduction of the required number of animal experiments as an alternative to the animal model.

However, there was a minor difference in ligand accumulation between PSMA^+^ and PSMA^−^ and a higher variation in the HET-CAM model compared with the mouse model.

In the mouse and especially the HET-CAM model, a minor accumulation was detected in the PSMA-negative PC-3 tumor. We obtained similar results in our studies with [^68^Ga]Ga-PSMA-11 [24]. Laidler et al. [49] demonstrated that under appropriate conditions, the expression of PSMA can also be re-established in PC-3. In our experiments, reconstituted expression of PSMA in PC-3 could not be noted in the Western blot and in the histological sections.

We hypothesize that the nonspecific accumulation in the PSMA-negative tumors results from the PSMA concentration in the blood. Blood retention seems to be prolonged in the chick model compared to the mouse model, which may explain the increased accumulation in the PC-3 tumor in ovo compared to the mouse. Since transient binding with albumin enables longer retention in blood for various compounds, it is also conceivable that a radioligand-albumin complex transiently reaches a higher concentration in the PSMA-negative tumor than in the blood itself due to the EPR effect. The same effect can also occur in the PSMA-positive tumors, but may be masked by the specific accumulation.

The TACs for the LNCaP C4-2 tumors show a steady increase over time in the in ovo and in the mouse model. Blood concentration (arterial input function) and continuous internalization and exchange of PSMA at the cell surface are the two dominant factors for the shape of the TAC. The steady increase in the TAC of LNCaP C4-2 in ovo is likely due to higher blood concentration over time, whereas in the mouse, the blood concentration decreases, causing a flattening of the curve over time.

Furthermore, the LNCaP C4-2 tumor in mice is significantly more vascularized and less hypoxic than the PC-3 tumor [40,41,42]. These anatomical differences between the tumors likely enhance the apparent PSMA^+^/PSMA^−^ ratio, which is why a further analysis of the vascularity of the tumors in the chick embryo model is needed.

Accumulation in the tumor is based on the combination of ligand binding and internalization. For PSMA, low temperatures of 4 °C, such as those occurring during cooling of the embryo, are known to stop internalization but have no effect on binding [50]. Therefore, an overall reduction of the accumulation due to the lowered body temperature cannot be completely ruled out. However, in the case of a complete absence of internalization, saturation of the binding sites will occur, causing a plateau in the TAC of LNCaP C4-2. Furthermore, PET measurements were started approximately 1.5 h after cooling, which we believe is sufficient time for rewarming of the chick embryo. A more detailed analysis may be provided by additional simulations in a PBPK model. If cooling affects internalization and pharmacokinetics of the ligand, alternative immobilization techniques such as isoflurane vaporization or application of liquid narcotics to the surface may be considered [45,51,52,53].

In the HET-CAM as well as in the mouse models, γ-counter measurements yield significantly higher activity concentrations than PET for LNCaP C4-2 (PSMA^+^) tumors. This may be caused by both an overestimation of the γ-counter due to residual blood in the investigated sample and an underestimation of the PET data due to partial volume effects (PVE). Therefore, in future experiments, the excess blood needs to be more accurately removed from the tumor tissue, e.g., by a specific perfusion protocol.

In addition, because tumor volumes were less than 1 mL in the HET-CAM model, overestimation of activity concentration might have played a role in both the γ-counter analyses and the PET evaluation. Small blood vessels in close proximity to tumors are often more difficult to remove and then contribute in γ-counter measurements. In the PET evaluation, such vessels adjacent to the tumor have an influence due to the PVE. The partial volume effect is already relevant for the comparatively large tumor and organ structures from mice and may be of particular importance due to the again significantly smaller tumor and organ volumes in the HET-CAM model.

The finite spatial resolution of the PET scanner leads to closely spaced anatomical structures with different activity levels in the image influencing each other. In these “spill-over” and “spill-in” effects, the activity of the region under consideration is underestimated or the activity in the region under consideration is overestimated due to activity from neighboring regions. In addition, there is the “tissue fraction effect” caused by the division of PET data into discrete voxels with activities often composed of a mixture of different anatomical regions, since voxels are not correlated with anatomy [54].

While partial volume correction in the chicken egg still requires an enormous effort, a PVE factor of small animal imaging can be optimized with less effort.

While positron range does not significantly matter at the 4-5 mm resolution of clinical scanners, the choice of radionuclide matters for PVE in studies using preclinical scanners, such as the Focus120 with an optimal spatial resolution of 1.13 mm full-width half-maximum (tangential, Filtered-Back-Projection) in the center field of view [46,55,56]. Comparing the mean positron range in water of ^68^Ga (2.9 mm) that was used in our previous study [24] and ^18^F (0.6 mm) [57,58,59], it is clear that ^68^Ga significantly reduces the overall resolution of the scanner. This problem is well known [60,61,62] and several approaches exist using partial volume correction [63], e.g., based on CT images [64] or direct calculations during image reconstruction [65] to solve the resolution problems. While there are already several approaches of partial volume correction using MR images [66,67,68], the application is still challenging.

Nevertheless, we consider the HET-CAM model to be a beneficial method for initial assessment of specific tumor binding of new compounds and, accordingly, for reducing animal testing.

### 4.2. Analysis Regarding the Applicability of the HET-CAM Model for Biodistribution Studies in Comparison to the Mouse Model

To the best of our knowledge, this work was the first to investigate comparability of radiopharmaceutical biodistribution in the chick embryo with the mouse model. Based on the imaging data, the key organs: heart, brain, liver, and kidney, may be identified. Spleen and lung could not yet be sufficiently differentiated by our current methods in the HET-CAM model. However, in future studies, it is expected to optimize the measurement methods to include these organs as well.

The biokinetics of the radioligand were successfully followed and quantitatively evaluated in the HET-CAM model. For the heart, liver, and brain, the data agreed well with the results of the mouse experiments.

In the kidneys, the highest activity concentration was also determined in the chick embryo model, but no increase in accumulation was detected over the measurement period.

The differences between the activity concentrations in the kidneys and those in the heart and liver after 10 min of the measurement period were significantly smaller in the HET-CAM model compared to the mouse model. The increased accumulation as well as the significantly slower decrease in activity concentrations in the kidneys compared to the liver and heart indicate that the signal decrease can only be explained to a minor extent by a decreasing ligand concentration in the blood pool.

The data based on heart, liver, and brain imply that the HET-CAM model can also be used for biodistribution analyses. The differences in kidney biodistribution require further analysis to allow a final evaluation and taking into account the influence due to the not yet fully developed organs and the distinct anatomical differences between avian and mammalian kidneys.

In humans, PSMA was detected in the proximal renal tubes of the renal cortex [69,70]. In mice, PSMA expression and specific binding of different ligands have been published [71,72,73,74]. Bacich et al. published analyses showing a 76% nucleotide and 86% amino acid homology between murine and human PSMA [75]. An alignment analysis between the protein sequences of human glutamate carboxypeptidase II (Q04609), and the homologous structures of mice (O35409) and corresponding putative protein in chicken (A0A1L1RPX5) revealed an amino acid equality between human and mouse PSMA of 84% and between human and chicken PSMA of 74.6%. Thereby, the amino acids for the binding regions, active region, and metal binding sites were highly conserved in all three proteins. However, no evidence of binding of PSMA-specific ligands to chicken PSMA has been published to date.

In the mouse model, the accumulation of [^18^F]F-siPSMA-14 in the renal cortex was clearly demonstrated visually. In general, the excretion of small molecules and peptides occurs via the kidneys [76,77]. While in the mouse, excretion occurs via the urinary bladder, in the closed system chick embryo, excretions are accumulated in the allantoic fluid [78]. No accumulation in the allantoic fluid has been observed (Figure S2).

According to Bolin et al. [79], kidney development in the chick embryo is advanced by EDD15, suggesting that the kidneys begin to function at this time. However, reduced accumulation can indicate incomplete functionality. Nevertheless, the reduced accumulation over time in the kidneys is consistent with extended blood retention.

Although mammalian and avian kidneys are structurally different, they share similar functions. In particular, avian kidneys possess both mammalian-type and reptilian-type nephrons that are equally active in adulthood [79,80,81]. Mammalian-type nephrons develop first during embryonic development. This development can be advantageous for the analysis of peptides for human application. Based on the present data, it cannot be demonstrated that PSMA is expressed in the embryonic avian kidneys or that corresponding radioligands can bind specifically. Studies of PSMA expression in avian kidneys may provide further information on the comparability of the models in the future.

The kinetics of activity in the heart is similar to the data from the mouse model. The chick embryo model allows analysis of the biodistribution of the radioligand. The signal in the heart region is usually based on the peptide concentration in the blood pool; no PSMA expression in the heart has been demonstrated thus far [70].

In PET, the signal in the heart was strongly influenced by the concentration in the blood; this was also observed when comparing PET and γ-counter data in mice. Here, the γ-counter signal was again weaker because the heart was rinsed during extraction.

The heart was already fully functional at the time of measurements [82]. The high activity concentration in the heart indicated a high blood concentration, and the elimination appeared to be slower than in the mouse.

In the liver in the chick embryo, similar kinetics to the mouse model were also observed. Again, our data confirm the suitability of the HET-CAM model for analyzing the biodistribution of radiolabeled peptides.

PSMA expression has not been previously demonstrated for liver [70]. The liver was already fully functional at the time of the analyses [83,84] and the decreasing kinetics suggest a nonspecific accumulation in the first minutes after injection.

The liver signal was also due to the concentration of the peptide in the vascular space as suggested by the strong correlation of the liver and heart data.

Analysis of biodistribution kinetics in the brain revealed interesting results. Although the brain, in particular the blood-brain barrier, was not yet fully developed at the time of analysis, only marginal accumulation in the brain was detected in the chick embryo, similar to the mouse model. Thus, the chick embryo model seems to be also applicable for the analysis of radioligands with respect to accumulation in the brain.

In detail, in both HET-CAM and the mouse model, the least activity concentration was detected in the brain.

In humans, expression of PSMA has been detected in the brain [85,86], but the blood-brain barrier prevents accumulation of PSMA-specific ligands. Similarly, in mice, an intact blood-brain barrier halts the accumulation of PSMA ligands [87,88,89]. Our data demonstrated that [^18^F]F-siPSMA-14 is also not transported across an intact blood-brain barrier in mice.

In the chick embryo, the blood-brain barrier is still developing. In the publications of Ribatti et al. and Roncali et al. [90,91], the permeability of the barrier was analyzed at different days of development with Evans Blue (960 Da) and with horseradish peroxidase (HRP, 40,000 Da), respectively. It was shown that after EDD14, HRP was no longer able to pass the barrier, whereas Evans Blue was detected until about 1 month after hatching. Evans Blue binds to serum albumin after application and then disperses as a complex [92,93]. In this form, it cannot cross the blood-brain barrier. Ribatti et al. postulated that during development, the serum albumin concentration was still low enough that free Evans Blue was present and could be transported across the blood-brain barrier.

The investigated ligand [^18^F]F-siPSMA-14, with a molecular mass of 1472.57 Da, corresponded more closely to the size of Evans Blue than HRP. Nevertheless, only marginal ligand accumulation could be detected, indicating minor transport across the blood-brain barrier. It is not known whether the ligand interacts with albumin.

In the mouse, an accumulation of the ligand in the spleen was also observed. This organ could not be differentiated in the chick embryo based on MR or PET imaging. Optimization of the MR scan may possibly reveal more details here in future studies.

Based on these data, the HET-CAM model may be potentially suitable for initial biodistribution analyses of peptide ligands in certain aspects; however, due to substantial obvious differences and limitations of the model, cannot fully replace further small animal studies in this respect.

### 4.3. Limitations

The HET-CAM model is limited in terms of metabolic processes due to the ongoing development of the chick embryo, which may differ from the adult animal and between different developmental stages. For example, the chick embryo undergoes strong metabolic changes during the different days of development. Hu et al. specifically studied gluconeogenesis, non-essential amino acid synthesis, and other citric acid cycle synthesis products at embryo development day 14 and EDD19 and demonstrated strong changes [94]. For this reason, when evaluating the HET-CAM model for biodistribution studies, it is important not only to detect the expression of the specific target structure of interest, e.g., glutamate carboxypeptidase II (PSMA), but to analyze it at different developmental stages.

Small anatomical structures with sizes smaller than three times the full-width half-maximum (FWHM) are affected by the PVE. Consequently, in the case of Focus 120, structures of 3.39 mm and smaller are affected [24,46], which include smaller tumors and small organs.

Tumor growth is primarily determined by the cell division rate. Due to the developmental days of the embryo as well as the planned experiments, the time for tumor growth is limited when working with cultured cells. There is also the possibility of growing tumor-like structures in vitro and then allowing them to grow into the CAM [95]. With this technique, it is also possible to establish larger tumors in a shorter time. Here, attention must be paid to vascularity.

For a small number of chick embryos, ratios of activity concentrations in the tumors (PSMA^+^/PSMA^−^) < 1 were determined using PET or γ-counter data. Again, tumor volume may have been a predominant factor. In most cases, PC-3 tumor volume was below average; thus, adjacent activities contributed more substantially to PVE. Large blood vessels in the immediate vicinity of the PC-3 tumor may lead to an overestimation of the signal in the tumor because of the spill-in/spill-over effects mentioned earlier. Volume determination was as accurate as possible based on MR images, but extraction of small tumor structures is significantly less accurate. Normalization to volume may cause portions of surrounding tissue with accumulated activity to cause an overestimation of actual tumor activity. To compensate for these inconsistencies, a larger number of chicken eggs may be required for reliable substance characterization. In Germany, the HET-CAM model is not considered an animal experiment. Thus, the larger number of chicken eggs (34 chick embryos versus 9 mice) corresponds to an animal saving in the sense of the 3Rs. For the future, a further reduction in the number of chick embryos will be envisaged by method optimization.

Accurate modeling of the kinetics of both tumor uptake and biodistribution to other organs requires continuous measurement from or shortly before the time of injection. Such PET measurements require catheter injection, which cannot be reliably performed in the chicken egg with the technical means available to us at the moment. The optimal approach here is to position the catheter by microsurgery assisted by a microscope, similar to what was conducted in 2013 by Warnock et al. [26]. We are committed to establishing these techniques in our laboratory in the future.

## 5. Conclusions

Our data indicate the high potential of the HET-CAM model to reduce the number of first-in-class animal experiments required for the development of new radiopharmaceutical with respect to the analysis of target specific binding. While our first data on analysis of biodistribution were promising, due to substantial differences of the chick embryo to adult small animals, further animal experiments in this context cannot be fully replaced.

## Figures and Tables

**Table 1:** Summary of the activity concentrations (mean ± standard deviation) in PSMA^+^ and PSMA^−^ tumor xenografts obtained with γ-counter and PET measurement for both in vivo models.

Prostate Cancer Cell Line	HET-CAM		Mice	
	γ-Counter [%IA/mL]	PET [%IA/mL]	γ-Counter [%IA/g]	PET [%IA/mL]
LNCaP C4-2 (PSMA^+^)	12.3 ± 6.4	2.8 ± 1.3	17.0 ± 10.5	9.4 ± 4.4
PC-3 (PSMA^−^)	9.3 ± 4.1	2.1 ± 1.3	1.8 ± 0.4	1.6 ± 0.4
Ratio [PSMA^+^/PSMA^−^]	1.5 ± 0.8	2.0 ± 1.5	9.5 ± 5.1	6.4 ± 3.2

**Table 2:** Summary of the mean activity concentrations in the organs of interest obtained with γ-counter and PET measurement for both in vivo models.

Organ	HET-CAM	Mice	
	PET [%IA/mL]	PET [%IA/mL]	γ-Counter [%IA/g]
Brain	1.8 ± 0.5	0.3 ± 0.1	0.1 ± 0.1
Heart	8.5 ± 2.0	2.6 ± 0.6	1.7 ± 0.4
Liver	7.4 ± 2.0	2.3 ± 0.5	1.7 ± 0.4
Kidneys	10.8 ± 4.2	47.0 ± 15.3	114.7 ± 34.2
(Eye)	1.8 ± 0.7	-	-
Spleen	-	12.1 ± 5.7	26.3 ± 15.3
Lung	-	2.1 ± 0.5	2.2 ± 0.6
Muscle	-	0.9 ± 0.2	0.8 ± 0.2
Bone	-	1.1 ± 0.3	1.5 ± 0.3
Blood	-	-	2.5 ± 0.9

## Data Availability

The used data, additional to those in the supplement, are available from the corresponding author on reasonable request.

## Supplementary Materials

The following are available online at https://www.mdpi.com/article/10.3390/cancers13164007/s1, Figure S1: Western blot of CAM tumor lysates. Figure S2: study on excretion into the allantoic fluid. Table S1: summary of activity concentrations in tumors of the HET-CAM model. Table S2: summary of activity concentrations in tumors of the mouse model. Table S3: summary of biodistribution data from the HET-CAM model. Table S4: summary of biodistribution data from the mouse model. Methods: description of the overlay procedure using PMOD.
