# Supplementary material for: Comparison of Quantification of Target-Specific Accumulation of [18F]F-siPSMA-14 in the HET-CAM Model and in Mice Using PET/MRI
Source: Cancers (Basel). 2021 Aug 9;13(16):4007. doi: 10.3390/cancers13164007 (PMC8393674; doi:10.3390/cancers13164007)
Supplement: Supplementary file 1 [file cancers-13-04007-s001.zip › cancers-1301835-supplementary.pdf]

Supplementary materials: Evaluation of the HET-CAM model for quantification of biodistribution and tumor accumulation of ^{18}F-siPSMA-14 using PET and MR imaging

Jessica Löffler, Carmen Hamp, Ellen Scheidhauer, Daniel Di Carlo, Christoph Solbach, Alireza Abaei, Li Hao, Gerhard Glatting, Ambros J. Beer, Volker Rasche, Gordon Winter

Table S1. Summary of activity concentrations in tumors of the HET-CAM model. Activity concentration in LNCaP C4-2 and PC-3 tumors including the ratios of activity detected in LNCaP C4-2/PC-3 based on γ-counter and PET and tumor volumes based on MRI in the HET CAM model

	**γ-counter [%IA/mL]**			**PMOD [%IA/mL]**			**Volume [mL]**	
**Chick embryo Nr.**	LNCaP C4-2	PC-3	Ratio LNCaP C4-2/PC-3	LNCaP C4-2	PC-3	Ratio LNCaP C4-2/PC-3	LNCaP C4-2	PC-3
**1**	7.47	4.66	1.60	5.02	4.30	1.17	0.041	0.030
**2**	15.75	4.67	3.37	3.82	2.45	1.56	0.028	0.038
**3**	2.46	2.26	1.09	2.64	0.66	3.97	0.033	0.026
**4**	19.72	7.99	2.47	4.50	2.20	2.04	0.018	0.017
**5**	26.89	19.30	1.39	2.35	0.72	3.27	0.015	0.016
**6**	10.95	8.59	1.27	1.46	0.50	2.93	0.026	0.011
**7**	6.77	8.04	0.84	1.56	0.36	4.28	0.032	0.012
**8**	26.32	9.94	2.65	6.12	3.31	1.85	0.027	0.033
**9**	19.11	10.54	1.81	3.26	3.86	0.85	0.021	0.025
**10**	19.47	8.18	2.38	4.51	2.91	1.55	0.024	0.043
**11**	11.89	3.94	3.01	3.28	1.45	2.27	0.033	0.047
**12**	11.13	7.80	1.43	1.77	1.72	1.03	0.024	0.018
**13**	6.59	8.21	0.80	4.38	3.95	1.11	0.017	0.014
**14**	14.77	13.42	1.10	2.73	1.22	2.24	0.017	0.018
**15**	6.49	12.87	0.50	2.59	0.40	6.47	0.012	0.003
**16**	10.46	8.10	1.29	2.96	1.64	1.80	0.015	0.008
**17**	13.01	10.58	1.23	1.19	2.31	0.52	0.031	0.027
**18**	16.41	13.81	1.19	0.54	1.94	0.28	0.026	0.024
**19**	8.55	12.10	0.71	2.53	3.26	0.78	0.018	0.016

**20**	12.94	12.17	1.06	2.48	0.61	4.06	0.025	0.017
**21**	7.36	13.55	0.54	2.11	4.02	0.53	0.029	0.012
**22**	5.75	12.72	0.45	1.18	3.98	0.30	0.014	0.045
**23**	8.83	5.11	1.73	2.58	1.50	1.72	0.037	0.019
**24**	18.33	14.86	1.23	2.76	1.45	1.91	0.026	0.019
**25**	6.66	2.78	2.40	3.55	2.20	1.62	0.027	0.028
**26**	5.53	6.68	0.83	1.81	1.34	1.35	0.015	0.025
**Mean**	**12.29**	**9.34**	**1.48**	**2.83**	**2.09**	**1.98**	**0.024**	**0.023**
**SD**	**6.42**	**4.14**	**0.79**	**1.31**	**1.26**	**1.45**	**0.008**	**0.011**
**Median**	**11.04**	**8.40**	**1.25**	**2.61**	**1.83**	**1.67**	**0.025**	**0.019**

Table S2. Summary of activity concentrations in tumors of the mouse model. Activity concentration in LNCaP C4-2 and PC-3 tumors including the ratio of activity detected in LNCaP C4-2/PC-3 based on γ-counter and PET and tumor volumes based on MRI in the mouse model.

	γ-Counter [%IA/g]			PET [%IA/mL]			Volumen [mL]	
**Mouse**	**LNCaP C4-2**	**PC-3**	**Ratio**	**LNCaP C4-2**	**PC-3**	**Ratio**	**LNCaP C4-2**	**PC-3**
**1**	39.03	2.21	17.69	18.74	1.63	11.47	0.102	0.145
**2**	10.60	1.78	5.94	7.55	1.83	4.12	0.389	0.36
**3**	16.88	1.42	11.90	7.35	1.54	4.76	0.132	0.383
**4**	20.14	1.52	13.22	10.08	1.34	7.53	0.213	0.127
**5**	10.30	2.36	4.36	8.22	1.68	4.88	0.6	0.163
**6**	8.45	2.04	4.14	5.27	2.03	2.60	0.131	0.383
**7**	13.58	1.50	9.05	8.31	0.89	9.30	0.327	0.138
**Mean**	**17.0**	**1.8**	**9.5**	**9.4**	**1.6**	**6.4**	**0.27**	**0.24**
**SD**	**10.5**	**0.4**	**5.1**	**4.4**	**0.4**	**3.2**	**0.18**	**0.12**
**Median**	**13.6**	**1.8**	**9.1**	**8.2**	**1.6**	**4.9**	**0.21**	**0.16**

Table S3. Summary of biodistribution data from the HET-CAM model. Activity concentration in different organs based on PET evaluation in the chick embryo.

Chicken embryo	Heart [%IA/mL]	Liver [%IA/mL]	Brain [%IA/mL]	Eye [%IA/mL]	Kidney [%IA/mL]
**1**	15.96	14.54	3.17	4.20	17.94
**2**	12.12	12.26	2.20	2.10	27.45
**3**	7.53	6.61	1.40	1.40	7.06
**4**	8.86	7.96	1.96	1.95	9.37
**5**	8.34	7.42	1.72	1.84	8.37
**6**	7.71	6.21	1.96	1.26	10.81
**7**	6.38	6.10	1.96	1.46	11.87
**8**	10.70	7.87	1.95	2.56	9.68
**9**	8.23	8.35	3.57	1.20	7.49
**10**	7.81	6.28	1.84	1.22	10.31
**11**	7.79	7.40	1.38	1.88	9.70
**12**	6.51	4.68	1.21	1.46	9.91
**13**	7.00	5.47	1.90	1.43	8.33
**14**	6.69	6.50	1.30	1.65	10.39
**15**	7.85	6.39	1.56	2.17	7.81
**16**	8.52	6.39	1.88	2.39	10.38
**17**	8.27	7.39	1.64	1.63	7.18
**18**	10.57	8.03	1.72	1.11	7.75
**19**	7.67	6.37	1.36	1.97	12.27
**20**	7.92	6.92	1.32	0.98	9.82
**21**	8.39	6.59	1.65	1.28	8.18
**22**	9.39	8.89	1.97	2.13	12.69
**23**	7.79	7.20	1.67	1.68	13.89
**24**	7.32	6.26	1.66	1.86	11.95
**25**	8.74	7.23	1.39	2.51	10.70
**26**	7.43	5.83	1.29	1.69	9.41
**Mean**	**8.52**	**7.35**	**1.79**	**1.81**	**10.80**
**SD**	**2.00**	**2.03**	**0.54**	**0.65**	**4.15**
**Median**	**7.88**	**6.76**	**1.70**	**1.69**	**9.87**

Table S4. Summary of biodistribution data from the mouse model. Activity concentration in different organs based on γ-counter and PET data in mice.

	γ-counter [%IA/g]									PET [%IA/mL]							
**Mouse**	**Brain**	**Heart**	**Liver**	**Kidney**	**Spleen**	**Lung**	**Muscle**	**Bone**	**Blood**	**Brain**	**Heart**	**Liver**	**Kidney**	**Spleen**	**Lung**	**Muscle**	**Bone**
**1**	0.19	1.99	1.82	92.79	22.09	1.65	0.77	1.15	3.00	0.27	2.84	2.30	31.72	11.99	2.39	1.03	1.22
**2**	0.26	1.94	1.71	136.51	29.19	2.74	0.80	1.08	2.82	0.31	2.97	2.28	55.00	10.16	2.28	1.15	1.30
**3**	0.14	1.36	1.19	62.66	11.49	1.88	0.45	1.34	2.05	0.27	1.98	1.74	26.36	2.22	1.72	0.55	0.98
**4**	0.13	1.03	1.37	167.89	58.94	1.59	0.62	1.47	1.55	0.32	2.31	2.12	65.34	13.12	1.85	1.07	0.87
**5**	0.16	2.34	2.27	132.05	22.20	3.27	0.96	1.59	4.19	0.44	3.72	3.31	63.17	14.84	2.94	1.16	1.32
**6**	0.12	1.94	1.97	101.00	20.18	2.03	1.10	1.72	2.49	0.26	2.71	2.41	49.34	10.72	2.35	1.02	1.50
**7**	0.03	1.58	1.42	109.90	20.09	2.18	0.87	1.85	1.72	0.29	1.91	2.01	38.32	21.38	1.45	0.68	0.66
**Mean**	**0.15**	**1.74**	**1.68**	**114.68**	**26.31**	**2.19**	**0.79**	**1.46**	**2.54**	**0.31**	**2.63**	**2.31**	**47.04**	**12.06**	**2.14**	**0.95**	**1.12**
**SD**	**0.07**	**0.44**	**0.38**	**34.17**	**15.29**	**0.61**	**0.22**	**0.28**	**0.91**	**0.06**	**0.63**	**0.50**	**15.29**	**5.74**	**0.50**	**0.24**	**0.30**
**Median**	**0.14**	**0.194**	**1.71**	**109.90**	**22.09**	**2.03**	**0.80**	**1.47**	**2.49**	**0.29**	**2.71**	**2.28**	**49.34**	**11.99**	**2.28**	**1.03**	**1.22**

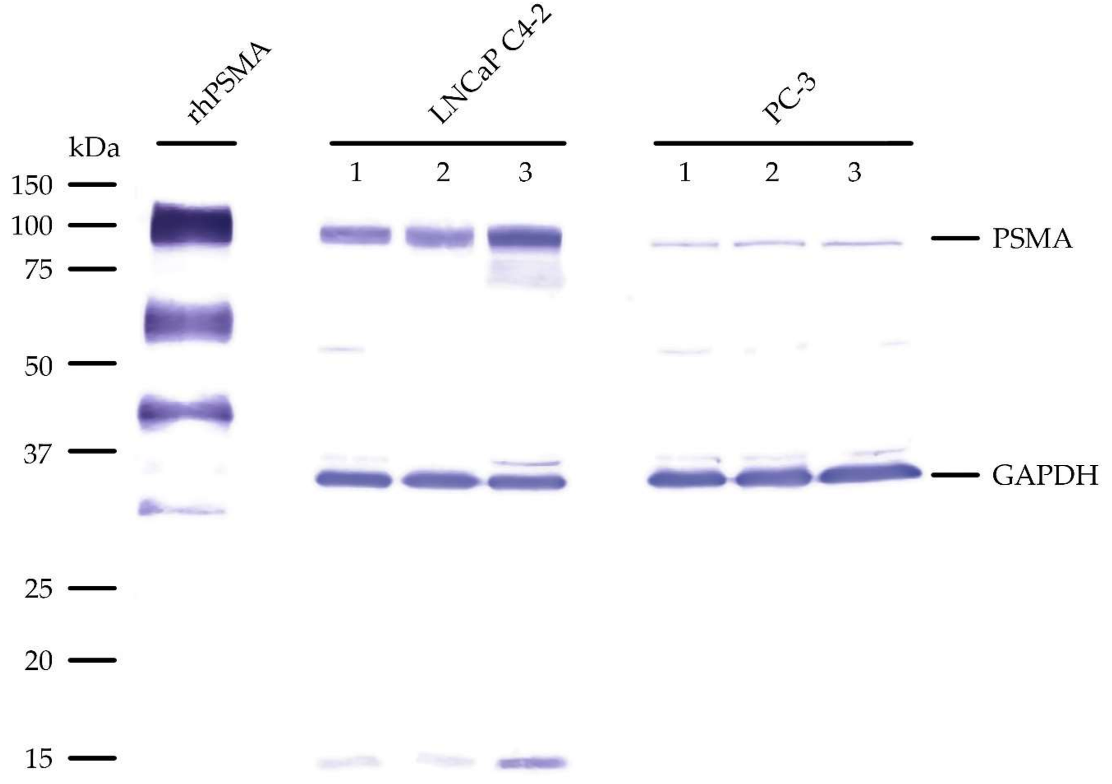

Figure S1. Western blot of CAM tumor lysates. Exemplary western blot of tumor-based lysates of LNCaP C4-2 and PC-3 derived from the HET-CAM model of three eggs to verify PSMA-expression.

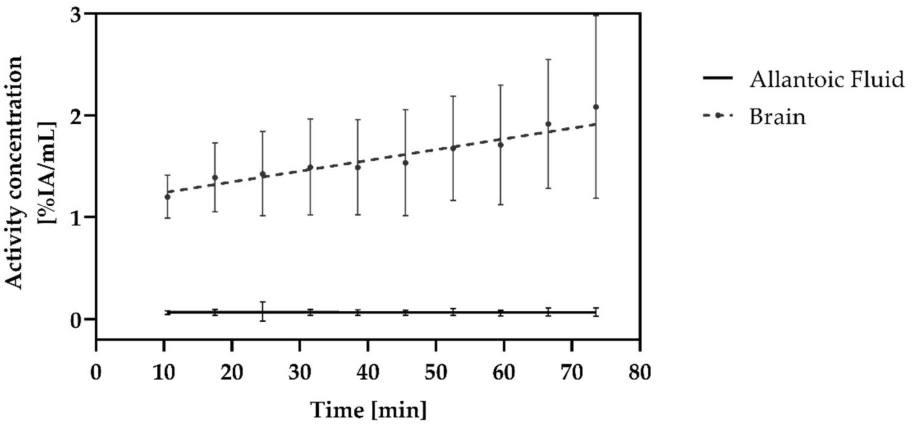

Figure S2: Study on excretion into the allantoic fluid. In the time-activity-curve the graphs of the simple linear regression for the allantois and in comparison for the brain are plotted. Only an extremely weak activity concentration was detected in the allantoic fluid, which also failed to produce any measurable change within the measurement period (slope: -0.00003 +- 0.00014).

Description of the overlay procedure using PMOD:

The PMOD program was used to overlay the PET and MRI data. For this purpose, the two data sets to be overlaid were loaded with the tool "Fuse-It". First, the input dataset was imported, in our case the PET dataset. The T1-weighted Flash 3D scan was used as a reference, since most VOIs were placed in this dataset and the reference dataset is not changed.

For optimal overlay, it was helpful to align both scans in the same orientation. To do this, the PET dataset is either adjusted after opening or an appropriate, previously saved, macro was applied during opening with the appropriate adjustment information.

When both scans were loaded, an automatic rigid overlay was performed. Normally, the program's overlay was of a good quality that it did not need to be corrected. In a few cases where correction of the overlay was necessary, the PET dataset was manually shifted or rotated.

A similar procedure was used for the overlay of the high-resolution T2-weighted RARE scan and the flash. Here, the RARE sequence was used as input and the flash once more as reference, and again an automatic rigid overlay was used. If necessary, the RARE scan was manually shifted or rotated slightly.
